# Resveratrol on the Metabolic Reprogramming in Liver: Implications for Advanced Atherosclerosis

**DOI:** 10.3389/fphar.2021.747625

**Published:** 2021-10-01

**Authors:** Ying Ma, Dongliang Li, Wenfeng Liu, Xiaoxiao Liu, Yingqi Xu, Xinrui Zhong, Fengnan Zhi, Xueling Jia, Yanan Jiang, Yuhua Fan

**Affiliations:** ^1^ Harbin Medical University-Daqing, Daqing, China; ^2^ Department of Pharmacology (State-Province Key Laboratories of Biomedicine- Pharmaceutics of China, Key Laboratory of Cardiovascular Research, Ministry of Education), College of Pharmacy, Harbin Medical University, Harbin, China; ^3^ Translational Medicine Research and Cooperation Center of Northern China, Heilongjiang Academy of Medical Sciences, Harbin, China

**Keywords:** resveratrol, atherosclerosis, liver metabolomics, lipid accumulation, metabolites

## Abstract

**Background/Aims:** Atherosclerosis (AS) is one of the major leading causes of death globally, which is highly correlated with metabolic abnormalities. Resveratrol (REV) exerts beneficial effects on atherosclerosis. Our aim is to clarify the involvement of liver metabolic reprogramming and the atheroprotective effects of REV.

**Methods:** ApoE-deficient mice were administered with normal diet (N), high-fat diet (H), or HFD with REV (HR). Twenty-four weeks after treatment, Oil Red O staining was used to assess the severity of AS. Non-targeted metabolomics was employed to obtain metabolic signatures of the liver from different groups.

**Results:** High-fat diet–induced AS was alleviated by REV, with less lipid accumulation in the lesions. The metabolic profiles of liver tissues from N, H, and HR groups were analyzed. A total of 1,146 and 765 differentially expressed features were identified between N and H groups, and H and HR groups, respectively. KEGG enrichment analysis uncovered several metabolism-related pathways, which are potential pathogenesis mechanisms and therapeutic targets including “primary bile acid biosynthesis,” “phenylalanine metabolism,” and “glycerophospholipid metabolism.” We further conducted trend analysis using 555 metabolites with one-way ANOVA, where *p* < 0.05 and PLS-DA VIP >1. We found that REV could reverse the detrimental effect of high-fat diet–induced atherosclerosis. These metabolites were enriched in pathways including “biosynthesis of unsaturated fatty acids” and “intestinal immune network for IgA production.” The metabolites involved in these pathways could be the potential biomarkers for AS-related liver metabolic reprogramming and the mechanism of REV treatment.

**Conclusions:** REV exerted atheroprotective effects partially by modulating the liver metabolism.

## Introduction

Atherosclerosis (AS) is a progressive metabolic disease characterized by an excessive accumulation of lipids in the arteries, which is the major contributor of coronary heart disease and stroke. In recent years, the relation between metabolic disorders and AS has been widely addressed (Aboonabi et al., 2019; Lin et al., 2020). Metabolic reprogramming contributes to the progression of AS (Vallée et al., 2019). The liver is an important primary metabolic organ of the body. The relationship between liver disorders and AS has been widely addressed, taking non-alcoholic fatty liver disease (NAFLD) as an example (Xin et al., 2020; Taharboucht et al., 2021a). NAFLD is significantly associated with AS, which provides an implication that the restoration of liver function may be useful in the management of AS (Taharboucht et al., 2021b).

Resveratrol (REV) is a natural polyphenol mainly present in plants belonging to *Vitis* L., *Veratrum* L., Arachis, Polygonum, etc. Many researchers evidenced the health perspectives of REV. In the past decade, REV has been proved to have extraordinary anticancer effects and cardiovascular protection abilities (Alrafas et al., 2020; Feng et al., 2020). REV has a profound prevention and therapeutic effect on AS (Berbée et al., 2013; Zhou et al., 2020). The effect of REV on metabolism has been uncovered. However, the effect of REV on human plasma lipid is controversial (Movahed et al., 2013; Sahebkar, 2013; Goh et al., 2014; Hausenblas et al., 2015; Sahebkar et al., 2015). These discrepancies among these studies might be due to the differences in study design, including the patient’s characteristics, REV dosage, and intervention duration. Recently, Akbari et al. further conducted a meta-analysis to evaluate the effects of resveratrol on the liver metabolism. They demonstrated that REV supplementation could reduce total cholesterol and increase gamma-glutamyl transferase (GGT) concentrations among patients with metabolic-related disorders (Akbari et al., 2020). In high-fat diet–fed mice, REV could reduce blood glucose, plasma triglyceride, and body weight and ameliorated insulin resistance (Gong et al., 2020). In hepatic cells, REV could reduce lipid accumulation and increase glycogen storage (Gong et al., 2020). Therefore, REV could also regulate metabolic reprogramming and thus exerts anti-atherosclerotic activity.

Even though a series of studies have been carried out, there is still a lack of direct evidence for the metabolic alteration of the liver in AS, and the involvement of metabolism in the anti-atherosclerotic activity of RSV. In the present study, we observed the metabolic reprogramming in AS and the involvement of liver metabolism in the anti-atherosclerotic activity of RSV. Liver tissues were collected from ApoE-deficient (ApoE^−/−^) mice from the standard chow diet (N), high-fat diet (H), and high-fat diet with REV-treated (HR) groups and analyzed using an untargeted metabolomics approach.

## Materials and Methods

### Animal Model Establishment and Treatment

ApoE^-/-^ mice (22 ± 2 g) were obtained from Nanjing Junke Biological Engineering Co., Ltd. Throughout the experiment, adequate food and water were provided. All procedures were approved by the Institutional Animal Care and Use Committee of Harbin Medical University [Protocol (2009)-11]. The use of animals was compliant with the Guide for the Care and Use of Laboratory Animals published by the U.S. National Institutes of Health (NIH Publication No. 85-23, revised 1996). All mice were randomly divided into normal (N), high-fat diet (H), and high-fat diet plus resveratrol (HR) groups. Mice in N, H, and HR groups were administered with standard chow diet (normal, N) or HFD (0.3% cholesterol and 21% (wt/wt) fat) for 24 weeks, respectively. REV (Sigma-Aldrich, Munich, Germany) was administered by oral gavage to the mice (10 mg/kg/day, twice a day) (Chen et al., 2018).

### Plaque Analysis

Twenty-four weeks after treatment, the en face aortas and aorta roots of mice were collected. The aforementioned samples were fixed with 4% PFA overnight and dehydrated with 30% sucrose. And then, the samples were embedded in OCT and frozen at −80°C (Chen et al., 2018). For the morphometric analysis, serial sections were cut into 6-μm-thickness slides using a cryostat. The sections were stained with hematoxylin and eosin (HE) for the quantification of the lesion area. Oil Red O staining was performed to indicate the lipid content in the lesions with an Oil Red O staining kit (Nanjing Jiancheng Biology Engineering Institute, Nanjing, Jiangsu, China) according to the manufacturer’s instructions. Aortic lesion size was obtained by averaging the lesion areas in four slides (12 sections) from the same mouse. The lesion area was analyzed as a percentage of the Oil Red O–stained area in the total aorta area. Every four slides from the serial sections were stained with HE, and each consecutive slide was stained with Oil Red O for the quantification of the atherosclerotic lesion area.

### Lipoprotein Profile and Lipid Analysis

The mice were fasted for 12–14 h before blood samples were obtained by retro-orbital venous plexus puncture. Before retro-orbital bleeding was conducted, the topical ophthalmic anesthetic—proparacaine—was applied. The study was conducted in compliance with the NIH Guide. Then plasma was collected by centrifugation and kept at −80°C. Total plasma cholesterol (TC), triglycerides (TG), high-density lipoprotein (HDL), and low-density lipoprotein (LDL) were enzymatically detected according to the manufacturer’s instructions (Nanjing Jiancheng Bioengineering Institute, China).

### Metabolite Extraction

Liver tissues from the mice were collected for metabolite extraction. The tissues were thawed on ice and grinded with liquid nitrogen. Then the metabolites were extracted (Huang et al., 2021). The repeatability and stability of LC-MS analysis were evaluated using the quality control (QC) sample. The QC samples were prepared by combining equal volume of each extraction. There are 12, 6, 7, and 5 samples in N, H, HR, and QC groups, respectively.

### LC-MS Analysis

All samples were analyzed using a TripleTOF 5600+ high-resolution tandem mass spectrometer (SCIEX, Warrington, United Kingdom) with both positive and negative ion modes. Chromatographic separation was conducted using an ultra-performance liquid chromatography (UPLC) system (SCIEX, United Kingdom). An ACQUITY UPLC T3 column (100 mm × 2.1 mm, 1.8 µm, Waters, United Kingdom) was applied for the reverse-phase separation. It was introduced for the separation of metabolites as the mobile phase consisted of solvent A (water, 0.1% formic acid) and solvent B (Acetonitrile, 0.1% formic acid). The gradient elution conditions were as follows, with a flow rate of 0.4 ml/min: 5% solvent B for 0–0.5 min; 5%–100% solvent B for 0.5–7 min; 100% solvent B for 7–8 min; 100%–5% solvent B for 8–8.1 min; and 5% solvent B for 8.1–10 min. The column temperature was maintained at 35°C. The TripleTOF Metabolites were measured by a high-resolution tandem mass spectrometer (TripleTOF 5600 Plus; SCIEX, Warrington, United Kingdom). To evaluate the stability of LC-MS, the QC samples were analyzed randomly.

### Metabolomics Data Processing

The acquired LC-MS data pretreatment was analyzed using XCMS software. Raw data files were converted into an mzXML format and then processed using the XCMS (Tautenhahn et al., 2012), CAMERA, and MetaX (Wen et al., 2017) in R software. Each ion was identified by the comprehensive information of retention time and m/z. The intensity of each peak was recorded. Then the information was matched to the in-house and public database including HMDB (http://www.hmdb.ca/), METLIN (http://metlin.scripps.edu/), Massbank (http://www.massbank.jp), PubChem (http://ncbi.nlm.nih.gov/) and KEGG (http://www.kegg.com/). Metabolites detected in ≥50% of QC samples or in ≥80% of total samples were collected. The missing data were extrapolated with the k‐nearest neighbor (KNN) algorithm. The data were processed by the probabilistic quotient normalization (PQN) algorithm and corrected by QC-robust spline batch correction (QC-RSC) using QC samples. Metabolic features with standard deviations ≤30% were collected.

### Trend Analysis

Metabolites with a one-way ANOVA *p* value < 0.05 and PLS-DA VIP >1 were selected to conduct trend analysis using the Short Time-series Expression Miner 1.3.11 (STEM). The metabolites altered in the H group were compared with N group and recovered by REV were collected.

### Statistical Analysis

The Student *t*‐test was used to compare the difference between two groups. *P* value was adjusted by using the Benjamini–Hochberg method. Principal component analysis (PCA) and partial least-squares discriminant analysis (PLS-DA) were conducted to identify differences between groups using MetaboAnalyst 3.0 (Xia et al., 2015). Features with a VIP >1.0 were collected. Two‐tailed Mann–Whitney *U* tests and two‐independent sample *t*-tests were performed using MeV 4.9.0 and PASW Statistics 18 software (SPSS Inc, Chicago, United States), respectively, to evaluate differences in metabolite levels (Saeed et al., 2006). Comparisons among three groups were performed using one-way ANOVA, followed by the Bonferroni post hoc test. A two-tailed *p* value < 0.05 was considered statistically significant. The data are expressed as mean ± SEM.

## Results

### Resveratrol Intervention Protected Against the Development of Atherosclerosis

**FIGURE 1:**
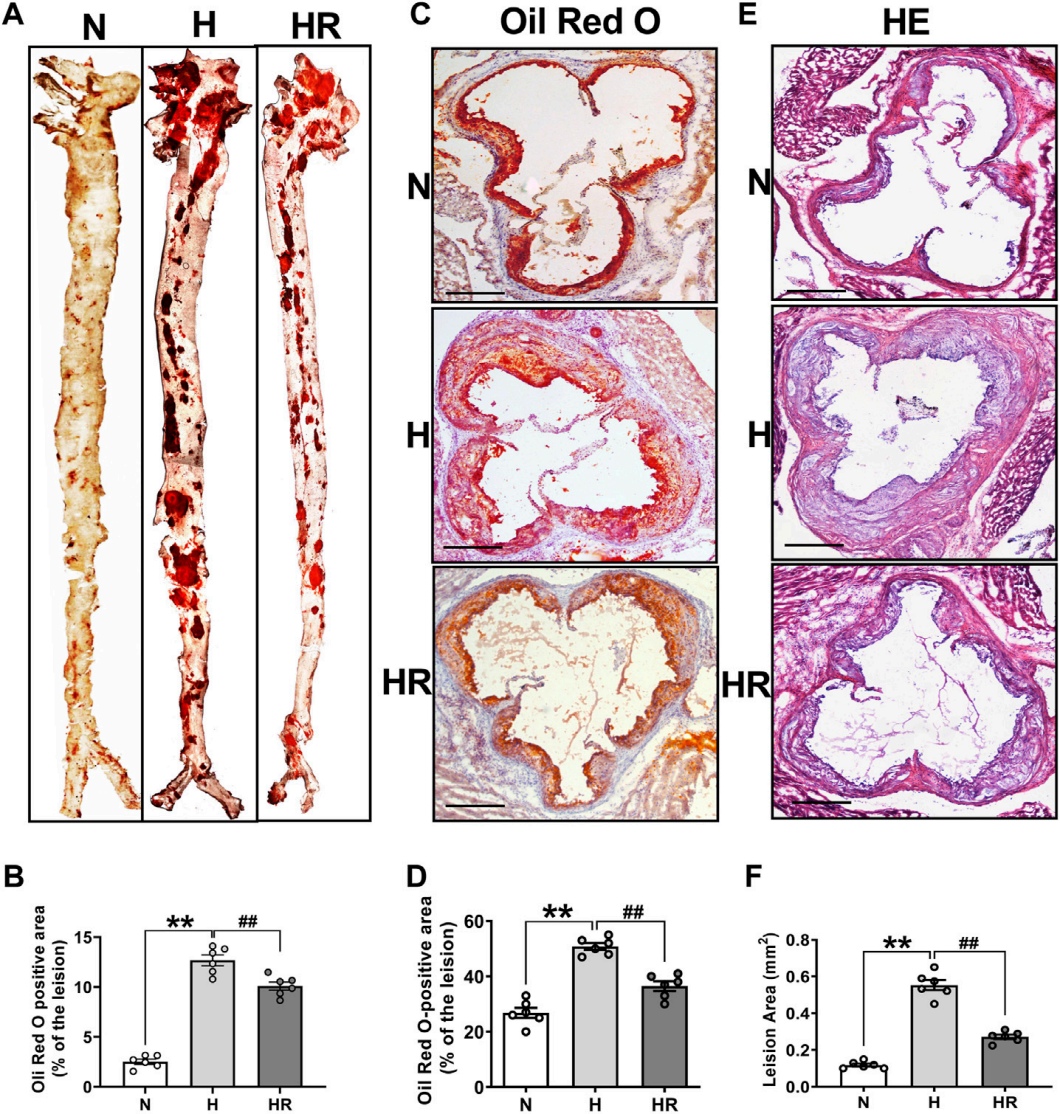
Resveratrol alleviated high-fat diet (HFD)–induced atherosclerosis in ApoE^−/−^ mice. **(A)** Representative images of en face aortas. **(C,E)** Oil Red O staining and HE staining of aortic root sections. Scale bar: 100 μm. **(B,D,F)** Quantification of lipid content and lesion size. *n* = 6 mice in each group. All data are the mean ± SEM. **p < 0.01 vs. *N* group; ^##^ *p* < 0.01 vs. HR group. “N” denotes normal; “H” indicates high-fat diet; “HR” denotes high-fat diet plus resveratrol (REV) treatment.

Oil Red O staining and HE staining were conducted to evaluate the effect of REV on atherogenesis. High-fat diet (HFD)–fed mice display more prominent features of atherosclerosis (AS), characterized by large atherosclerotic plaques and lipid accumulation in the lesions. However, resveratrol (REV) treatment arrested diet-induced AS that was observed from en face aortas and aortic roots (Figures 1A,C). The quantitative analysis was also conducted and showed its corresponding bottom values (Figures 1B,D). Next, HE staining was also performed to validate the anti-atherosclerotic effect of REV (Figures 1E,F). These results were in accordance with our previous findings that REV possesses anti-atherosclerotic activity (Ye et al., 2019).

### Resveratrol Ameliorated the Deteriorated Serum Lipid and Liver Lipid Accumulation During Atherogenesis

**FIGURE 2:**
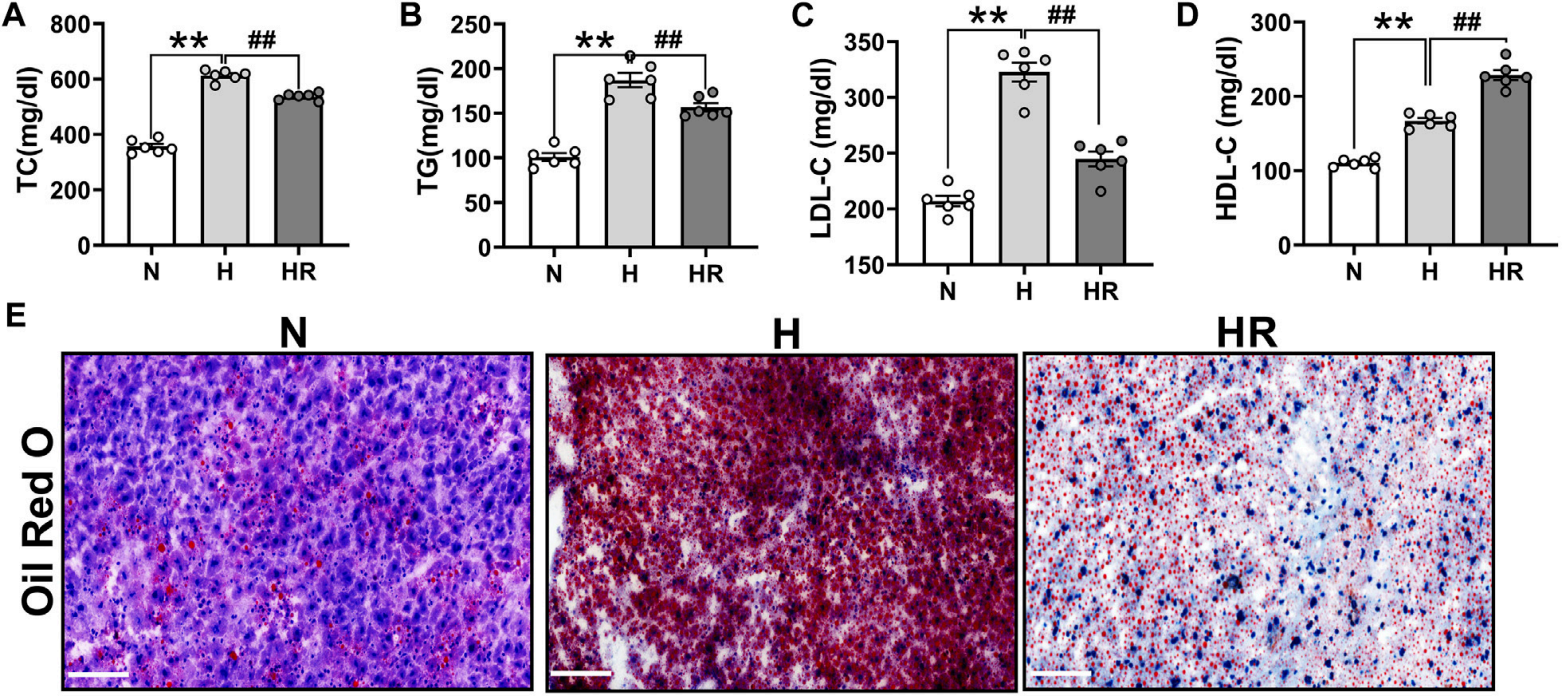
Resveratrol regulated serum lipid and prevented the lipid accumulation in the liver of ApoE^−/−^ mice. **(A–C)** Total cholesterol (TC) **(A)**, triglycerides (TG) **(B)**, and low-density lipoprotein cholesterol (LDL-C) **(C)** were dominantly inhibited by treatment with REV for 24 weeks in ApoE^−/−^ fed with HFD. **(D)** REV could increase the level of high-density lipoprotein (HDL) in ApoE^−/−^ fed with HFD. **(E)** Representative examples of liver tissues stained by using Oil Red O. ×200, Scale bar. *n* = 6 mice in each group. All data are mean ± SEM. **p < 0.01 vs. N group; ^##^ *p* < 0.01 vs. HR group. “N” denotes normal; “H” indicates high-fat diet; and “HR” denotes high-fat diet plus resveratrol (REV) treatment.

Anomalies of serum lipid levels are the main reason for atherogenesis. We observed that long-term administered resveratrol (REV) could obviously reduce the plasma levels of total cholesterol (TC) (Figure 2A), triglycerides (TG) (Figure 2B), and low-density lipoprotein cholesterol (LDL-C) (Figure 2C) and dominantly improve high-density lipoprotein (HDL) (Figure 2D) in the ApoE^-/-^ mice fed with HFD. Moreover, REV decreased liver lipid accumulation in HFD-fedafter treatment with REV in the liver of ApoE^-/-^ mice (Figure 2E).

### Metabolomics Analysis of Liver Samples by LC-MS

**FIGURE 3:**
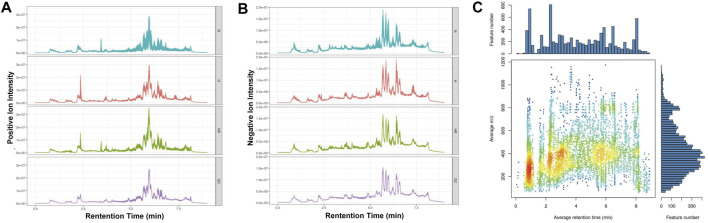
Quality control of metabolomics detection. **(A)** The total ion chromatogram (TIC) of all liver samples in the positive model. **(B)** Total ion chromatogram (TIC) of all liver samples in negative model. **(C)** The m/z–rt distribution. Normal (N); high-fat diet (H); and high-fat diet plus resveratrol (HR).

The metabolic profiles of liver tissues from the three groups were analyzed by LC-MS. The quality of detection was analyzed by XCMS software. The total ion chromatogram (TIC) showed the separation of all metabolites in the UPLC. In both the ESI+ and ESI- model, there are differences among these groups, indicating that HFD and REV treatment affected liver metabolite expression (Figures 3A,B). Whether these altered metabolites are involved in the pathogenesis and REV treatment of AS needs further analysis. In mass spectrometry, each substance has specific m/z and rt (Figure 3C).

### The Quantification of Metabolites

**FIGURE 4:**
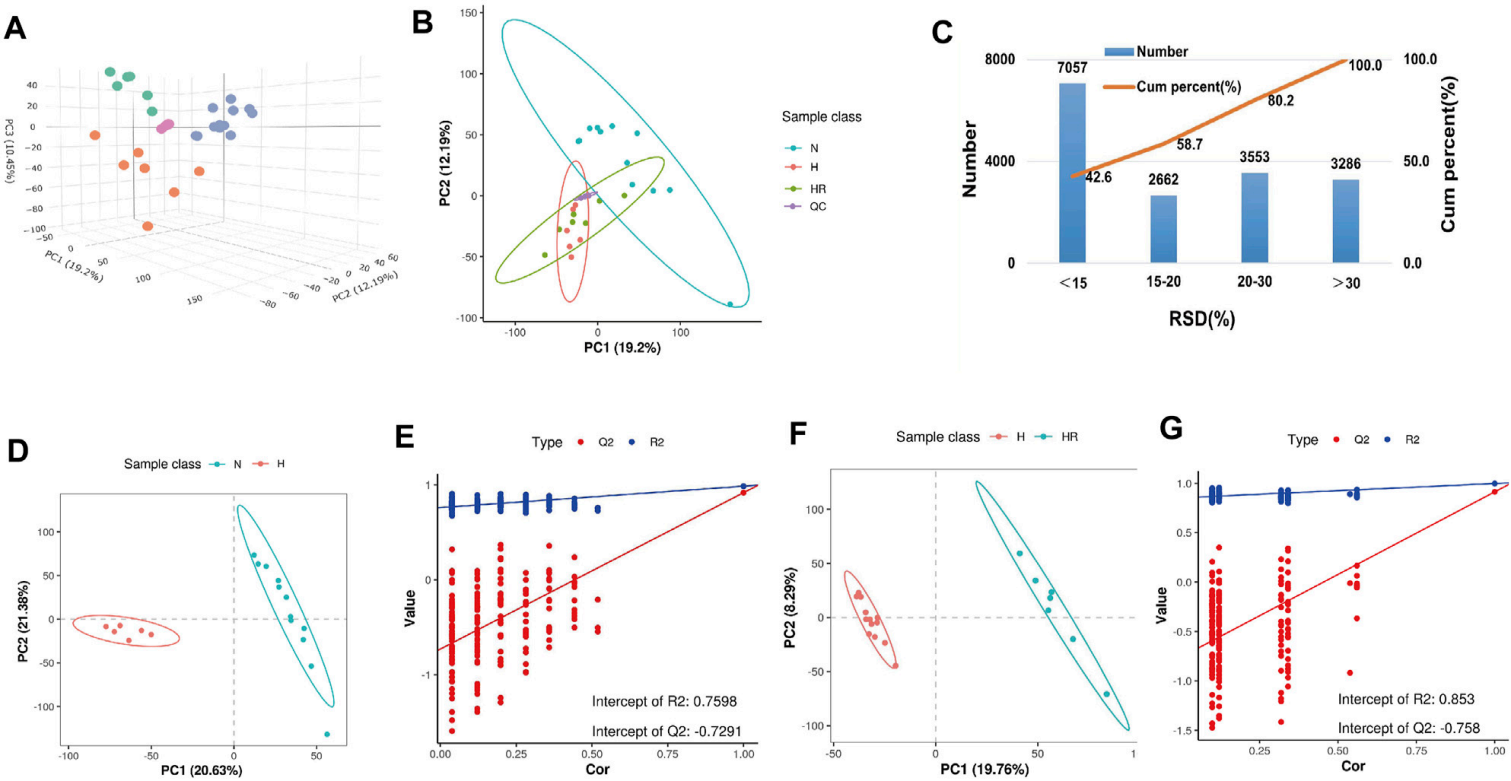
Metabolic profiling analysis of liver samples. **(A)** Three-dimensional score plot of the samples using the PCA model. **(B)** Two-dimensional score plot of the samples using PCA model. **(C)** Relative standard deviation (RSD) distributions of ions in QC samples. **(D,F)** Score plot of samples from normal (N), high-fat diet (H), and high-fat diet plus resveratrol (HR) groups using the PLS-DA model. **(E,G)** Corresponding validation of PLS-DA of N and H groups **(D)**, and H and HR groups **(F)**.

The PCA showed the different variables among the 3 groups, indicating that the three groups exhibited obviously different metabolites (Figures 4A,B). QC samples exerted high reproducibility with each other. The total ions were acquired from the QC samples. Among the total 16,558 ions obtained from the QC samples, the relative standard deviation (RSD) value of 9,719 ions (58.7% of total ions) was less than 20% (Figure 4C). PLS-DA was performed to further delineate the metabolic differences among the three groups (Figures 4D–G). The data distribution between the high-fat diet (H) and normal (N) groups (Figures 4D,E), as well as high-fat diet (H) and high-fat diet plus resveratrol (HR) groups was different (Figures 4F,G), respectively.

### 3.5 Differentially Expressed Metabolite Analysis

**FIGURE 5:**
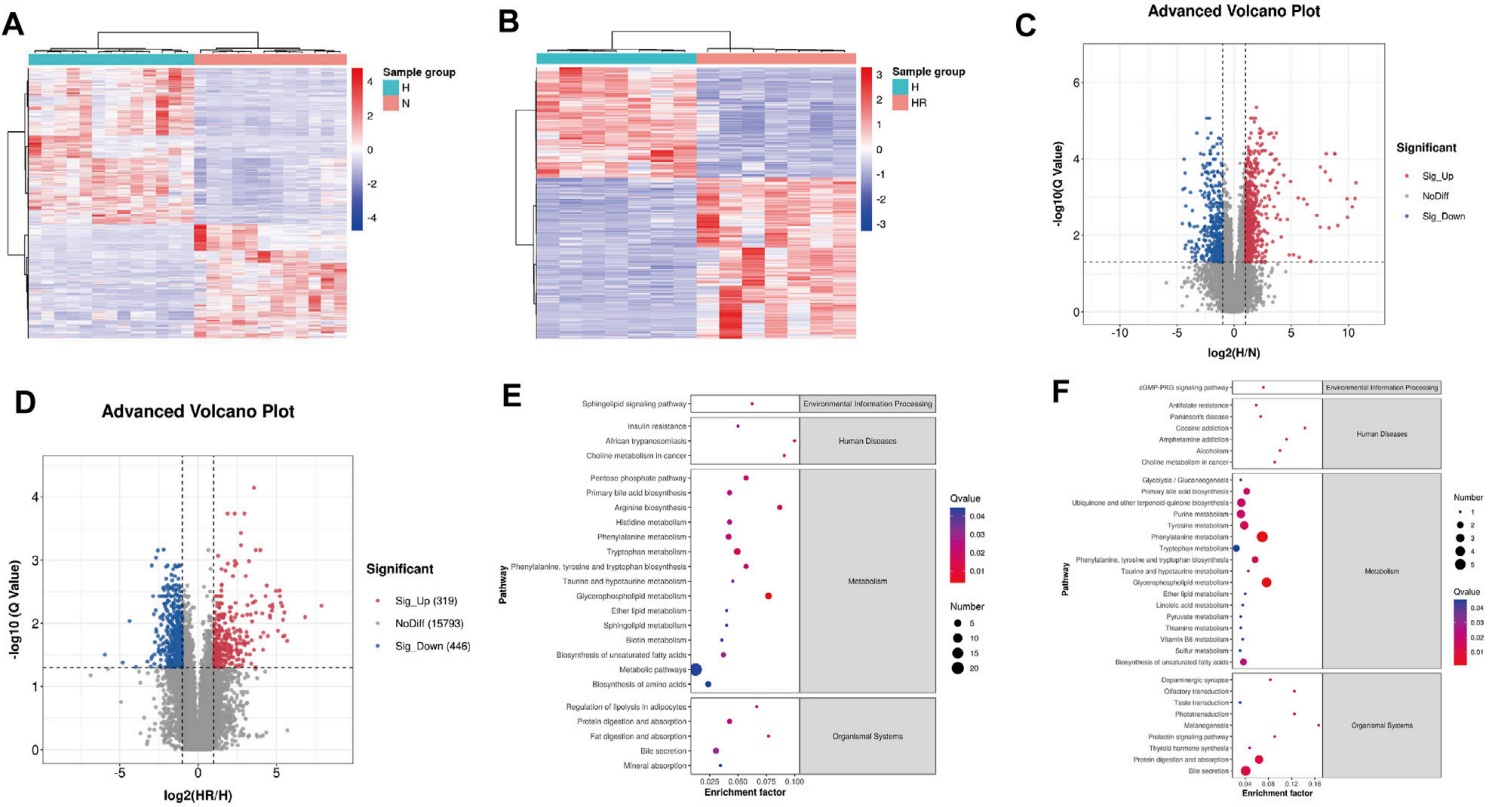
Differential metabolites screened by metabolomics analysis and metabolic pathway analysis. **(A)** Heat map of differentially expressed metabolites between N and H groups. **(B)** Heat map of differentially expressed metabolites between H and HR groups. **(C)** Volcano plots of differentially expressed metabolites between N and H groups. **(D)** Volcano plots of differentially expressed metabolites between H and HR groups. **(E)** KEGG enrichment of differentially expressed metabolites between N and H groups. **(F)** KEGG enrichment of differentially expressed metabolites between H and HR groups. N, normal group; H, high-fat diet group; and HR, high-fat diet plus REV treatment group.

The heat map and volcano plot revealed different metabolite profiles between N and H groups, as well as H and HR groups (Figures 5A–D). Differentially expressed features were identified as |log2 (fold change)| ≥ 1, Q ≤ 0.05, and VIP ≥ 1. A total of 1,146 and 765 differentially expressed features were identified between N and H groups, as well as H and HR groups, respectively (Figures 5A–D; Table 1).

**TABLE 1:** Number of differentially expressed metabolites between groups.

Sample	Neg-all	Neg-down	Neg-up	Pos-all	Pos-down	Pos-up	All regulated
H/N	8,757	179	401	7,801	218	348	1,146
HR/H	8,757	257	161	7,801	189	158	765

Then the KEGG pathway analysis was also performed. It was shown that the differentially expressed metabolites between N and H group were enriched in pathways including “glycerophospholipid metabolism,” “metabolic pathways,” and “phenylalanine metabolism” (Figure 5E). And the differentially expressed metabolites between H and HR groups was enriched in pathways including “phenylalanine metabolism,” “glycerophospholipid metabolism,” and “primary bile acid biosynthesis” (Figure 5F). There are many overlapped pathways between N/H and H/HR; these pathways are potential pathogenesis mechanisms and therapeutic targets, especially metabolism-related pathways including “primary bile acid biosynthesis,” “phenylalanine metabolism,” and “glycerophospholipid metabolism.”

### Trend Analysis of Differentially Expressed Metabolites

**FIGURE 6:**
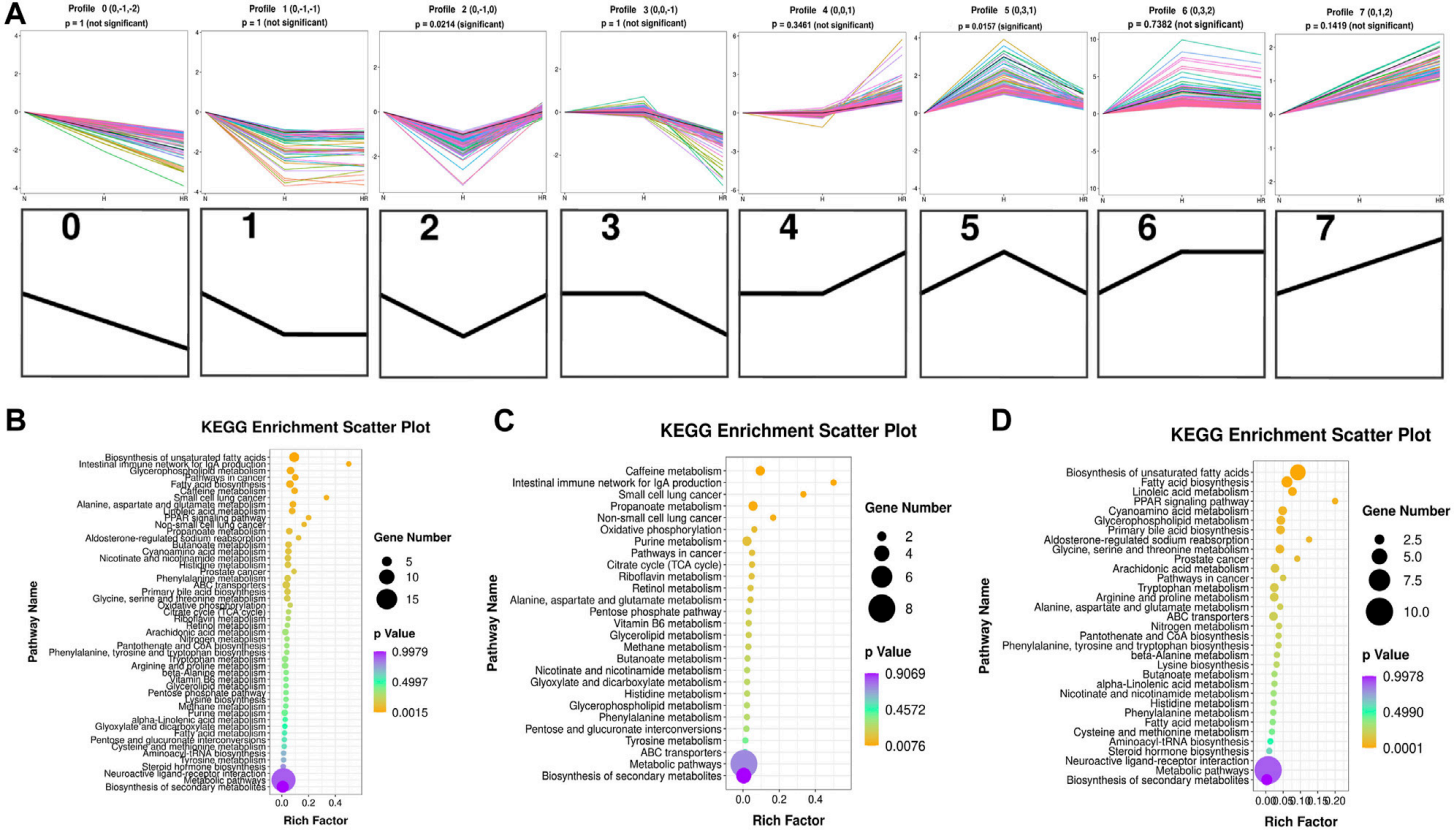
Trend analysis of differentially expressed metabolites. **(A)** Expression trend of metabolites. All the distinct color lines represented different second metabolites. The top seven figures indicated the trend of all different metabolites. And the bottom seven black figures denoted the trend profile. **(B)** KEGG enrichment scatterplot of trends 2 and 5. **(C)** KEGG enrichment scatterplot of trend 2. **(D)** KEGG enrichment scatterplot of trend 5. N, normal group; H, high-fat diet group; and HR, high-fat diet plus REV treatment group. Rich factor indicates the number of differentially expressed genes located in the KEGG/the total number of genes located in the KEGG. The smaller the *p* value, the higher the concentration of KEGG.

Five hundred fifty-five metabolites with a one-way ANOVA *p* < 0.05 and PLS-DA VIP >1 were selected to conduct trend analysis using STEM software. The trend analysis images represent trends in metabolites across the multiple comparison groups, and each small image represents one trend. Metabolites in trends 2 and 5 were selected, and KEGG enrichment was performed using MBRole 2.0 (http://csbg.cnb.csic.es/mbrole2/index.php) (Figure 6A). Metabolites in trend 2 were downregulated by HFD and recovered by REV. On the contrary, metabolites in trend 5 were upregulated by HFD and recovered by REV. The top four enriched pathways of metabolites in trends 2 and 5 were “biosynthesis of unsaturated fatty acids,” “intestinal immune network for IgA production,” “glycerophospholipid metabolism,” and “pathways in cancer” (Figure 6B). The top four enriched pathways of metabolites in trends 2 were “caffeine metabolism,” “intestinal immune network for IgA production,” “small-cell lung cancer,” and “propanoate metabolism” (Figure 6C). And the top four enriched pathways of metabolites in trend 5 were “biosynthesis of unsaturated fatty acids,” “fatty acid biosynthesis,” “linoleic acid metabolism,” and “aldosterone-regulated sodium reabsorption” (Figure 6D).

### Potential Biomarkers for Liver Metabolism

**FIGURE 7:**
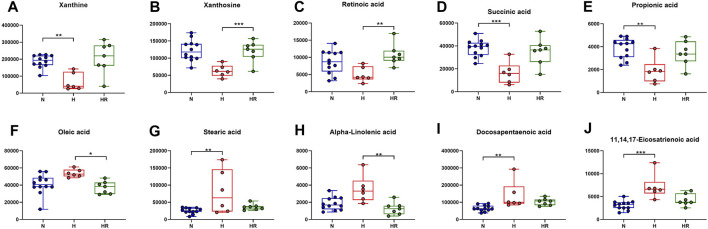
Effects of REV on potential biomarkers for liver metabolites. **(A–E)** Expression of xanthine **(A)**, xanthosine **(B)**, retinoic acid **(C)**, succinic acid **(D)**, and propionic acid **(E)**. **(F–J)** Expression of oleic acid **(F)**, stearic acid **(G)**, alpha-linolenic acid **(H)**, docosapentaenoic acid **(I)**, 11,14,17-eicosatrienoic acid **(J)**. N, normal group, *n* = 8; H, high-fat diet group, *n* = 10; and HR, high-fat diet plus REV treatment group *n* = 7. All data are mean ± SEM. *p < 0.05 N vs. H group; ^#^ *p* < 0.05 HR vs. H group, two-tailed Mann–Whitney *U* test.

Based on the KEGG analysis results, the metabolites in pathways with high significance were selected. Xanthine, xanthosine, retinoic acid, succinic acid, and propionic acid were increased in the liver of mice with high-fat diet, whereas REV reversed the expression of these metabolites (Figures 7A–E). On the contrary, some metabolites were decreased in the liver of mice with high-fat diet, the expression of which was reversed by REV, including oleic acid, stearic acid, alpha-linolenic acid, docosapentaenoic acid, and 11,14,17-eicosatrienoic acid (Figures 7F–J). These metabolites are potential biomarkers for liver metabolic reprogramming induced by HFD.

## Discussion

Increasing evidence reveals that liver metabolic disturbances are the dominant inducers of atherogenesis. In the present study, an untargeted metabolomics approach was employed to investigate liver metabolic perturbation during AS and the atheroprotection activity of REV.

In this study, we validated that REV treatment alleviated HFD-induced AS in ApoE^−/−^ mice (Figure 1). Besides, REV could also reduce TC, TG, and LDL-C levels in serum, as well as lipid accumulation in the liver (Figure 2). Liver metabolism disorder was considered as an important inducer of AS. To detect the effect of REV on HFD-induced metabolite alteration in the liver, the liver tissues of mice were collected to detect the alteration of metabolites by untargeted metabolomics. The total ion chromatogram and m/z–rt distribution showed that the detection quality was high (Figure 3).

As shown in Figure 4, an obvious difference was observed between N, H, and HR groups based on PCA and PLS-DA. With a cutoff value of |log2 (fold change)|≥ 1, Q ≤ 0.05, and VIP≥ 1, a total of 1,146 and 765 differentially expressed features were identified between N and H groups, and H and HR groups, respectively (Figures 5A–D; Table 1). KEGG enrichment analysis was further performed between N and H groups, and H and HR groups. In comparison with chow diet–fed mice, HFD altered 35 pathways (Figure 5E). In addition, REV treatment altered 42 pathways (Figure 5F). Our result found that “primary bile acid biosynthesis,” “phenylalanine metabolism,” “glycolysis/gluconeogenesis,” “pentose phosphate pathway,” “pyruvate metabolism,” and “sulfur metabolism” were all involved in both atherosclerosis progression and the prevention of REV. These findings suggested that REV could alter multiple metabolic pathways in the liver and thus exerts atheroprotection activity (Figures 5E,F).

The plasma trimethylamine-N-oxide (TMAO) level was elevated in AS patients (Randrianarisoa et al., 2016). TMAO could promote the progress of AS by modulating cholesterol and sterol metabolisms (Koeth et al., 2013). In ApoE^−/−^ mice, TMAO could accelerate aortic lesion formation through decreasing hepatic bile acid synthesis (Ding et al., 2018). However, REV could attenuate AS by decreasing TMAO levels and increasing hepatic bile acid synthesis (Chen et al., 2016).

We further performed trend analysis using differentially expressed metabolites (one-way ANOVA P< 0.05 and PLS-DA VIP>1) (Figure 6A). Metabolites in trend 2 and/or 5 may have diagnosis and therapeutic potential, which were enriched in pathways including “biosynthesis of unsaturated fatty acids,” “intestinal immune network for IgA production,” “glycerophospholipid metabolism,” “caffeine metabolism,” and “biosynthesis of unsaturated fatty acids.” Metabolites were involved in the biosynthesis of unsaturated fatty acids (oleic acid, stearic acid, alpha-linolenic acid, docosapentaenoic acid, and 11,14,17-eicosatrienoic acid), fatty acid biosynthesis (oleic acid, stearic acid, and myristic acid), linoleic acid metabolism (alpha-dimorphecolic acid and 12,13-dihydroxy-9Z-octadecenoic acid), and aldosterone-regulated sodium reabsorption (alpha-dimorphecolic acid) were increased in HFD-fed mice (Figures 6, 7), whereas metabolites involved in caffeine metabolism (xanthine and xanthosine), intestinal immune network for IgA production (retinoic acid), and propanoate metabolism (succinic acid and propionic acid) were decreased in mice with HFD (Figures 6, 7).

Abnormal lipid metabolism has been considered as a major mechanism in the development of atherosclerosis. Unsaturated fatty acids were reported to increase biomarkers for atherosclerosis in obese and overweight non-diabetic patients (de Oliveira et al., 2017). For fatty acid biosynthesis–related metabolites, increased liver oleic acid synthesis was observed in the liver of cholesterol-fed rabbits (Sivaramakrishnan and Pynadath, 1982). Oleic acid also could induce fatty live models *in vitro* (Okamoto et al., 2002). Moreover, a high circulating oleic acid level is a risk factor for atherosclerosis (Steffen et al., 2018). This was in accordance with our findings that HFD could increase the expression of oleic acid, which was attenuated by REV. Besides, in our experiment, retinoic acid was decreased in the liver of high-fat diet–fed mice. It was reported that retinoic acid could prevent the development of atherosclerosis in mice (Jiang et al., 2008). These findings were in accordance with our results. Therefore, the pro-AS factor, HFD, induces liver metabolic reprogramming. REV treatment exerts an anti-atherosclerotic effect, at least partially through the alteration of critical metabolites in the liver. However, there are still some metabolites with uncertain effects in AS.

## Conclusion

This research clarified the therapeutic effects and underlying mechanisms of REV for treating AS from the perspective of liver metabolomics. We found that metabolites and related pathways were altered in the liver from the diet-induced AS mouse model. REV reversed some of these metabolites and pathways in the liver, which might be a potential mechanism for atheroprotection.

## Data Availability

The original contributions presented in the study are included in the article/supplementary material; further inquiries can be directed to the corresponding authors.

## Abbreviations

ApoE^−/−^ mice, ApoE-deficient mice; AS, atherosclerosis; GGT, gamma-glutamyl transferase; H group, high-fat diet group; HR group, high-fat diet with REV-treated group; KNN algorithm, k‐nearest neighbor algorithm; NAFLD, non-alcoholic fatty liver disease; N group, normal diet group; PCA, principal component analysis; PLS-DA, partial least-squares discriminant analysis; PQN algorithm, probabilistic quotient normalization algorithm; QC-RSC, QC-robust spline batch correction; REV, resveratrol; TIC, total ion chromatogram.
